# Screening and treatment practices for tuberculosis infection in Nordic, Baltic and Central European countries and Ukraine in 2023

**DOI:** 10.1007/s10096-026-05471-y

**Published:** 2026-03-18

**Authors:** Thijs Feuth, Iiris Rajalahti, Mikko Vauhkonen, Karine Nordstrand, Melanie Stecher, Piret Viiklepp, Elmira Gurbanova, Ülla-Karin Nurm, Yana Terleeva, Tuula Vasankari

**Affiliations:** 1 Finnish Lung Health Association, Helsinki, Finland; 2 Department of clinical medicine, Faculty of Medicine, University of Turku, Turku, Finland; 3 Department of lung diseases and allergology, Turku University Hospital, Turku, Finland; 4 Department of lung diseases and allergology, Tampere University Hospital, Tampere, Finland; 5 Department of Infectious Disease Control and Vaccines, Norwegian Institute of Public Health, Oslo, Norway; 6 Estonian Tuberculosis Register, Department of Registries, National Institute for Health Development, Tallinn, Estonia; 7 Department of Pulmonary Medicine, University of Tartu, Tartu, Estonia; 8 Northern Dimension Partnership in Public Health and Social Well-being, Stockholm, Sweden; 9 Tuberculosis Management and Counteraction Department, Public Health Center of the MOH of Ukraine, Kyiv, Ukraine

**Keywords:** Tuberculosis, Tuberculosis infection, Latent tuberculosis infection, Screening, Prevention, MDR-TB

## Abstract

**Purpose:**

Throughout Europe, tuberculosis (TB) remains a public health concern, particularly among high-risk groups. Identifying at-risk populations for tuberculosis infection (TBI) testing and treatment is one of the tools to control the TB epidemic. This study aims to assess policies and practices in tuberculosis infection management across 10 countries in the Nordic, Baltic and Central European region, collaborating through the Northern Dimension Partnership in Public Health and Social Well-being network (NDPHS), and Ukraine.

**Methods:**

National data on TB epidemiology, management and policies were collected through an online questionnaire, followed by structured interviews with country representatives. Data were verified and meaningful similarities and differences were identified through follow-up contacts and working group discussions.

**Results:**

Among NDPHS countries, TB incidence ranged from 3 to 22 per 100,000 population in 2023, with multidrug resistance in 4.1% (441/10855) of cases. In NDPHS countries, Ukrainians accounted for 46.4% (189/407) of multidrug-resistant cases. While all countries screen for TBI among immunosuppressed patients and TB contacts, approaches to refugee screening and preventive treatment vary, 5 of 11 countries lacked access to rifapentine. Most countries did not utilize levofloxacin in the preventive treatment of multidrug-resistant TBI. Two countries reported having a national registry for TBI.

**Conclusion:**

Significant variation exists in TBI management across countries. Optimizing screening and treatment strategies directed at populations at risk is crucial for TB control in low-incidence countries. Cross-border coordination could be improved through alignment with international guidelines and by establishing an international registry for TBI.

**Supplementary Information:**

The online version contains supplementary material available at 10.1007/s10096-026-05471-y.

## Introduction

Whereas tuberculosis (TB) is still among the top ten causes of death worldwide, TB incidence in the World Health Organization (WHO) European Region has steadily dropped to 24 cases (range 21–28) per 100,000 population, representing 2.1% of the total global burden of TB [1, 2]. In many European countries, the majority of new TB cases is diagnosed in migrants from high-incidence countries, while a history of higher incidence rates in the past is reflected by predominance of the elderly among native patients, who may develop disease decades after primary infection [3]. 

Tuberculosis infection (TBI), defined by an individual’s persistent immune response to TB without evidence of TB disease, is estimated to be prevalent in around 24% of the world population and in 14% in the WHO European Region [4, 5]. The condition, which was previously termed ‘latent tuberculosis infection’, may result in TB disease in around 5–10% of individuals [2]. TBI can be treated to prevent TB disease, which is especially of importance in patients at high risk of developing active disease, such as household contacts or those with weakened immunity. In the 2024 updated guideline on tuberculosis preventive treatment (TPT), WHO proposes screening for TBI in risk groups with either a tuberculin skin test (TST) or interferon-y release assay (IGRA). Treatment options after exposure to drug sensitive (DS) TB are six to nine months of treatment with isoniazid or three months with isoniazid combined with rifampicin with daily dosing or high-dose isoniazid combined with rifapentine once a week. Additionally, rifampicin for three to four months or a combination of isoniazid with rifapentine for one month daily are possible regimens to be used. The new WHO recommendation of six months of daily levofloxacin to contacts exposed to multidrug- or rifampicin-resistant tuberculosis (MDR/RR-TB) was released in the same guideline [6]. 

The incidence of TB and the prevalence of TBI vary over countries and between specific groups at risk, while the incidence within these risk groups may also be dynamic over time [7]. Screening and treatment practices for TBI vary between different countries and settings [8, 9, 10]. This may reflect specific epidemiological conditions, resources and other factors.

Within the WHO European region, the burden of TB and MDR-TB is highest in eastern Europe and in countries on the Asian continent, such as the Russian Federation and Central Asia [2]. The current war in Ukraine’s territory, with subsequently millions of individuals seeking refuge across European countries, poses complex challenges in TB control to these countries, in many of which rates of TB are typically low [11, 12]. Ukraine had a relatively high incidence of TB already before the full-scale invasion in 2022, with drug- resistant patterns in more than 30% of cases [11, 12]. In Germany, 597 of 4677 refugees from Ukraine (13%) had a positive IGRA screening result, while three (0.1%) had active TB, including one with MDR-TB [13]. In a survey among 43 countries, the TB notification rate was 12.8 per 100,000 migrants born in Ukraine [14]. Norway is one of few European countries requiring universal screening for TB among all refugees. TB notification rates among newly arriving Ukrainian refugees increased to 104.0 per 100,000 in 2024, driven by a 3-fold increase among adult males since 2022 [15]. 

In this study, we map the screening and treatment practices of TBI in European countries that collaborated through the Northern Dimension Partnership in Public Health and Social Well-being (NDPHS) as well as Ukraine, to identify challenges and best practices in the management of TBI.

## Materials and methods

All countries of the NDPHS were invited to participate in this study, and Ukraine was included because of its current priority. NDPHS is a partnership of countries in the Nordic and Baltic region, aimed at international expert-level cooperation to promote sustainable and inclusive public health.

To map the treatment and screening practices among NDPHS countries and Ukraine, we developed a survey based on discussions with all partners. The study aimed to collect quantitative and qualitative data related to 2023 TB epidemiology and the management of TBI. The online survey was drafted by two investigators using Surveypal (Surveypal Oy, Finland) and revised based on feedback from the other investigators. The survey was shared with country representatives from the relevant national institutes, which were identified in the project meetings. For Ukraine, the questionnaire was shortened to match with the current country-specific conditions. For Latvia, some data that was unavailable from 2023 was supplemented with 2022 data. After initial data analysis and identification of possible inaccuracies, the informants were contacted to verify or correct the information provided.

Based on the results from the questionnaire, topics and structured questions for additional clarifying interviews with the country representatives were identified. These interviews were performed via video calls with individual country representatives, except for Lithuania (because of no response) and Ukraine (to minimize extra workload during full-scale war against Ukraine), and also explored challenges and good practice tips, as well as attitudes towards the possibility of an international TBI registry. Based on the interviews, a list of 18 candidate parameters for an international TBI registry was proposed, and country representatives were asked to vote for up to 10 of these parameters in a second, post-interview survey (Supplementary Material).

The summaries of the country interviews were shared among the investigators and findings were discussed in video meetings with the authors (YT excepted) to identify similarities and differences between the countries, and to relate the findings to international guidelines and the latest evidence from the literature [6, 16, 17]. 

The final, descriptive analysis with totals and proportions of the corrected data was performed in Microsoft Excel, and bar graphs were created with Microsoft PowerPoint. Missing data was accounted for in the statistical analyses, and this was always clarified in the text and tables. The epidemiological data from Ukraine is presented separate from that of NDPHS countries. For the evaluation of screening practices, data from Ukraine were integrated with data from NDPHS countries when appropriate.

## Results

### Participating countries

Data was obtained from informants of 10 NDPHS countries (Denmark, Estonia, Finland, Germany, Iceland, Latvia, Lithuania, Norway, Poland, Sweden) and Ukraine.

## Incidence of TB disease in NDPHS countries

Epidemiological data on TB disease from 2023 was obtained from all 10 NDPHS countries. From one country, only core data was available from 2023 and was hence supplemented with data from 2022. TB incidence varied from 3 per 100,000 inhabitants in Denmark, Finland, Norway and Sweden to 22 per 100,000 in Lithuania. Patients with MDR-TB made up 441 of 10,855 TB patients (4.1%), ranging from 0 of 16 TB patients (0%) in Iceland and 6 of 361 (1.7%) in Sweden to 10 of 78 patients (12.8%) in Estonia. The proportion of foreign-born TB patients ranged between 25% and 88% in countries with low TB incidence (< 10 per 100.000) and between 4% and 9% in countries with incidence above 10 per 100,000 population.

Within NDPHS countries, individuals from Ukraine made up 543 of 9,922 patients with DS-TB (5.5%) and 187 of 400 patients with MDR-TB (46.8%) (data from Denmark and Latvia excluded due to incomplete data). The number of HIV-TB co-infected patients was not available from Germany and Denmark due to legal constraints. In other countries, people living with HIV (PLWH) made up 102 of 6066 total TB cases (1.7%), ranging from 0.0% in Iceland and 0.7% in Poland to 12.8% in Estonia. The epidemiologic data are presented in Table 1.

**Table 1:** Tuberculosis epidemiology

	Incidence of new TB		New MDR-TB		New pre-XDR and XDR TB	New TB in < 16 years old		HIV-TB		foreign born	Ukrainians with new TB excluding MDR		Ukrainian migrants among new MDR	
	*N*	/100.000	*N*	% of new TB		*N*	% of new TB	*N*	% of new TB	% of new TB	*N*	%	*N*	% of new MDR
Denmark	186	3	7	3.8	1	4	2.2	n.a.	n.a.	25	n.a.	n.a.	2	28
Estonia	78	6	10	12.8	0	3	3.8	10	12.8	25	7	10.3	3	30
Finland	178	3	12	6.7	2	3	1.7	9	5.1	52	13	7.8	6	50
Germany	4481	5	184	4.1	48	242	5.4	n.a.	n.a.	77	292	6.8	92	50
Iceland	16	4	0	0.0	0	1	6.3	0	0	88	0	0	0	-
Latvia^1^	347	15 #	34	9.8	3	6 #	2.1 #	17	4.9	9 #	1 #	0.4 #	2 #	6 #
Lithuania	619	22	72	11.6	14	37	2.2	20	3.2	4	12	2.2	5	7
Norway	153	3	15	9.8	4	7	4.6	5	3.3	87	25	18.1	12	80
Poland	4436	12	101	2.3	10	51	1.1	32	7.2	8	189	4.4	68	67
Sweden	361	3	6	1.7	1	11	3.0	9	2.5	85	5	1.4	1	17
**All NDPHS**	**10,855**	**n.a.**	**441**	**4.1**	**83**	**359 §**	**3.4 §**	**102 ^**	**1.6 ^**	**n.a.**	**543 ¥**	**5.4 ¥**	**191 §**	**46.4 §**
Ukraine	19,851	48	3549	17.9	910	639	3.2	3350	16.9	n.a.	-	-	-	-
**All**	**30,706**	**n.a.**	**3990**	**13.7**	**993**	**998 §**	**9.2 §**	**3452 ^**	**13.3 ^**	**n.a.**	**-**	**-**	**-**	**-**

# Latvia data from 2022; § Latvia excluded due to incomplete data; * Ukraine and Denmark excluded; ^ Denmark and Germany excluded due to incomplete data; **¥** Denmark and Latvia excluded due to incomplete data; ECDC: European Centre for Disease Prevention and Control;

*DS-TB* drug sensitive tuberculosis, *HIV-TB* tuberculosis in individuals with human immunodeficiency virus infection, *MDR* multidrug resistant tuberculosis, *n.a.* data not available, *TB* tuberculosis, *XDR* extensively drug-resistant tuberculosis

## TB epidemiology in Ukraine

Ukraine reported 19,851 new TB cases in 2023, which accounted for 65% of all cases reported in this study, including 3,549 cases of MDR-TB, accounting for 17.9% of new TB cases in Ukraine, and 89% of MDR-cases in this report. Of those, 910 cases were extensively drug-resistant tuberculosis (XDR-TB) or pre-XDR-TB, accounting for 4.6% of new TB cases in Ukraine and 91.6% of (pre-)XDR-TB in this report. 3.2% of new TB cases in Ukraine were diagnosed in individuals below 16 years old, and 16.9% of new TB cases were diagnosed in PLWH. The epidemiologic data is presented in Table 1.

## Screening practices of TB and TBI in NDPHS countries and Ukraine

National guidance for TBI practices is included in the national TB guidelines in six countries and is under planning in four countries. Two countries had separate guidelines for specific risk groups, while the absence of TBI guidelines was reported in one country.

Among the WHO recommended TBI screening groups, contacts of smear positive pulmonary TB cases are screened for TBI in 10 out of 11 countries (excluding Poland). In all 11 countries, people initiating anti-tumour necrosis factor (TNF) treatment were offered screening. In most countries, PLWH (in 9/11 countries), people preparing for transplantation (9/11), dialysis patients or with end-stage renal disease (7/11) are also routinely screened for TBI. Screening of patients with silicosis is routinely performed in only 5/11 countries. Other groups such as contacts of smear negative pulmonary TB patients (6/11), and refugees and asylum seekers aged up to 15 years (6/11) are systematically screened for TBI in some countries.

**Fig. 1:**
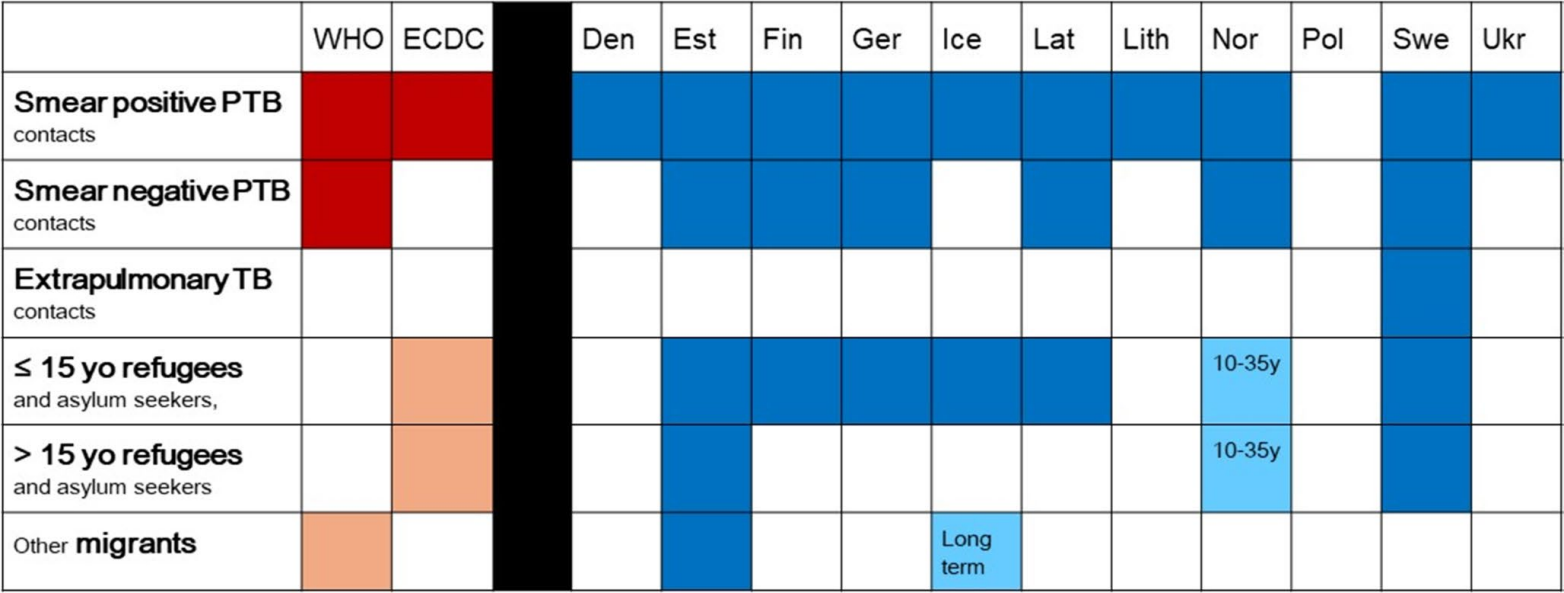
General risk groups TBI screening practices in NDPHS countries and Ukraine. Routine screening for TBI per risk group as utilized in Denmark, Estonia, Finland, Germany, Iceland, Latvia, Lithuania, Norway, Poland, Sweden and Norway (blue), and as proposed by WHO and ECDC (red). WHO and 2018 ECDC guidelines optional risk groups are marked red. Marked light blue or light red marked are conditional or optional

**Fig. 2:**
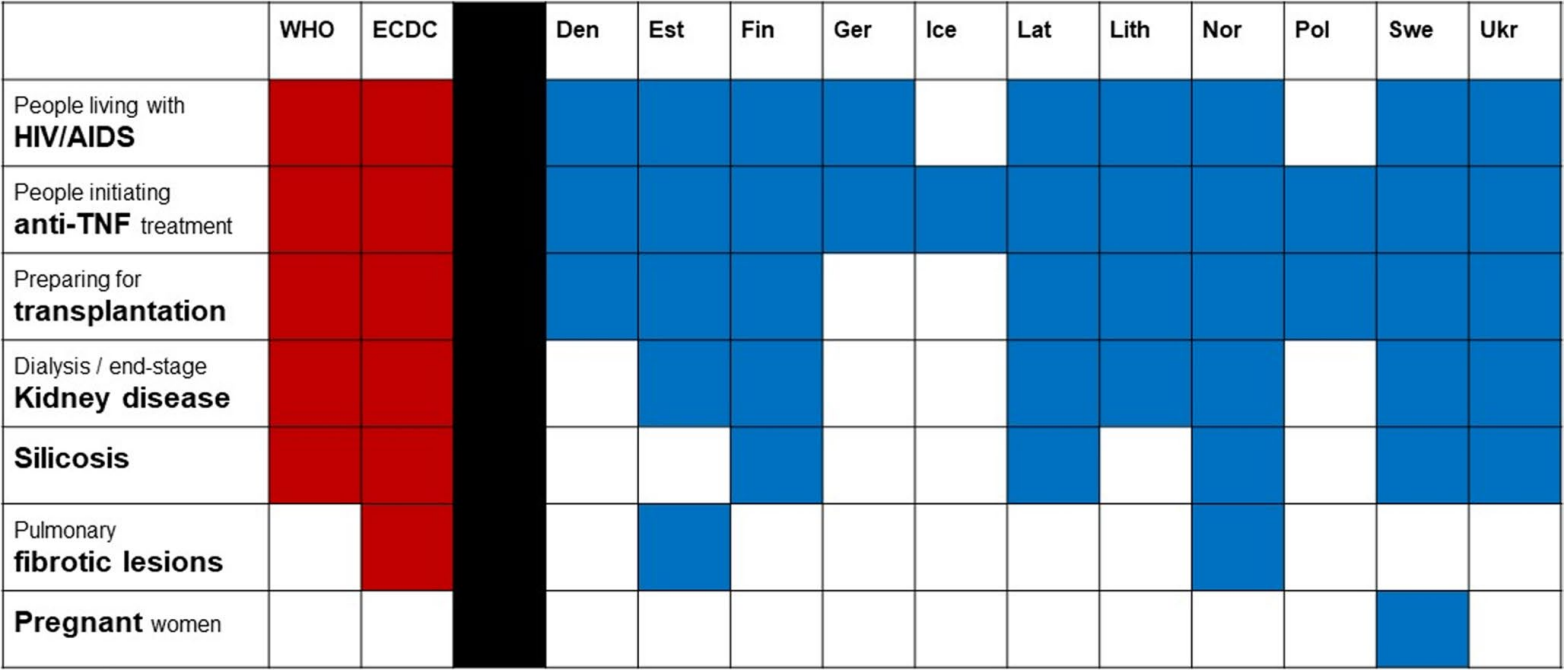
Medical risk groups TBI screening practices in NDPHS countries and Ukraine

In three countries, mandatory testing is in place under specific conditions. In Germany, screening for TBI is mandatory in contacts of smear positive as well as smear negative TB patients. Mandatory screening for pulmonary TB disease in refugees and asylum seekers and/or other migrants was also reported by Germany, while in these individuals, federal states may require preceding screening by IGRA in those under 15 years of age and/or in pregnant women. In Norway, testing is mandatory for contacts of smear-positive as well as smear-negative pulmonary TB, as well as in refugees, asylum seekers and other migrants from particularly high-incidence (> 200 per 100,000 population) countries, depending on their age (< 35 years) and expected duration of stay (> 3 months). In Iceland, testing for TBI is mandatory for long-term immigrants, while those applying for a residence permit undergo mandatory testing for pulmonary TB disease. The utilization of screening among risk groups is depicted in Figs. 1 and 2.

In 4 out of 10 NPDHS countries, Ukrainian migrants are systematically offered screening for TBI. For this, IGRA was used in all four of these countries, Mantoux was also used as alternative option in two countries.

The IGRA QuantiFERON^®^ was documented as the primary method to detect TBI in 10 of 11 countries and Mantoux in two (with IGRA and Mantoux evenly in Ukraine). Mantoux test was used in remote areas or where IGRA is not available (in two countries); in children (in five countries, with specific criteria applied differently in each country); or when screening before planned immunosuppression in combination with IGRA in one country.

### Treatment of TBI in NDPHS countries

**Fig. 3:**
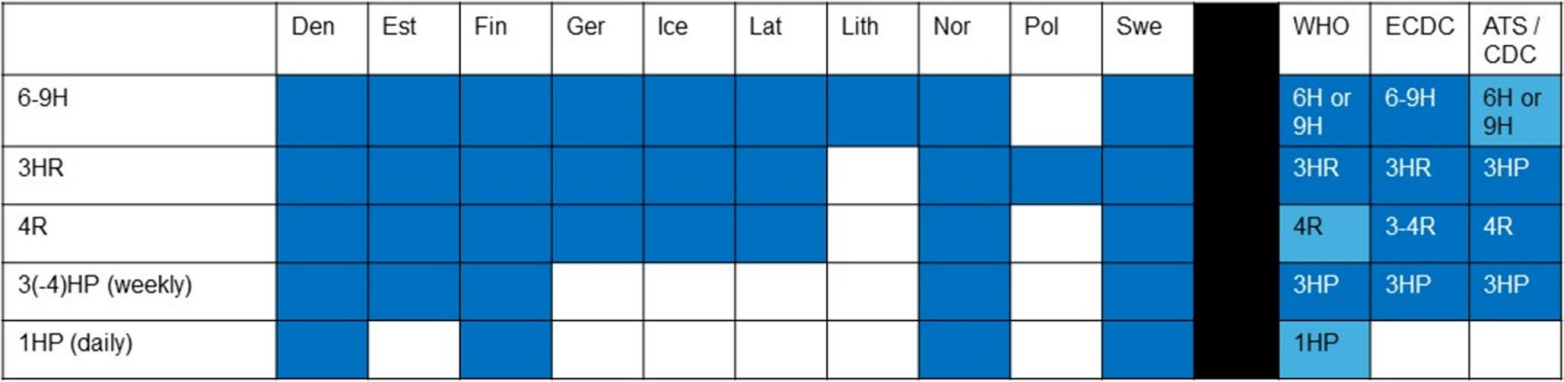
TPT regimens utilized in NDPHS countries. Utilization of TPT regimens for assumed drug sensitive cases in NDPHS countries Denmark, Estonia, Finland, Germany, Iceland, Latvia, Lithuania, Norway, Poland and Sweden. Dark blue: standard utilized or recommended, light blue: alternative treatment options according to the 2024 WHO guidelines and the ATS/CDC/ERS/IDSA guidelines and 2018 ECDC guidelines. 6–9 H: six to nine months of isoniazid, 3 h: 3 months of isoniazid and rifampicin; R rifampicin alone, 3 HP: 3 months of weekly rifapentine and isoniazid; 1HP one month of daily rifapentine and isoniazid

The treatment regimens for TBI included daily isoniazid for 6 to 9 months (9/10 NDPHS countries), daily isoniazid and rifampicin combination for 3 to 4 months (9/10 countries), daily rifampicin alone for 4 months (8/10), isoniazid and rifapentine once weekly for 3 months (5/10), and isoniazid and rifapentine daily for 1 month (4/10). Rifapentine was reportedly not available in 5/10 countries. The utilization of TPT regimens is presented in Fig. 3.

Eight countries reported that TBI in individuals exposed to MDR -TB may be subject to TPT and in one country, these patients are not treated (data lacking from 2 countries). In these patients, the treatment decision may depend on the drug sensitivity profile and on patient factors such as the risk of developing active TB disease. Drugs utilized for TPT after exposure to MDR-TB were fluoroquinolones (5/8 countries), delamanid (2/8), and/or ethambutol (1/8). One country did not report which drugs were used in such cases.

In the ten NDPHS countries, TPT may be initiated and monitored in specialised health care (nine countries), by TB services (four countries) or in primary healthcare (one country). In cases of treatment by a specialist, this may involve a pulmonologist (in six countries), an infectious diseases specialist (in five), a paediatrician (in four), a rheumatologist (in one) or a dermatologist (in one country).

Once initiated, TPT is monitored through directly observed treatment (DOT) or video-supported treatment (VST) in Finland (for rifapentine-isoniazid regimens) and Latvia (all TBI except for those treated with isoniazid), through weekly or monthly observation in Estonia and Lithuania or a mix of approaches including DOT and VST in Norway. Iceland reported telephone or in-person intermittent review, with DOT rarely applied. In four countries, observation is not routinely applied (Denmark, Germany, Norway and Sweden). Monitoring routinely includes blood tests such as blood count, creatinine, alanine transaminase, and alkaline phosphatase in seven countries. Data on TPT initiation and monitoring was not available from Ukraine.

## Documentation and registration of TBI

Use of International Classification of Diseases (ICD) codes for TBI was inconsistent and included Z20.1 (contact with and exposure to tuberculosis) in four countries, Z22.7 (carrier [suspected] of tuberculosis) in four, and Z03.0 (observation for [suspected] tuberculosis), Z22.3 (carrier of other specifies bacterial diseases), and R76.1 (Mantoux, abnormal result) in one country. Only Norway and Latvia reported having a national registry or surveillance in place for TBI, in both countries, only covering individuals who initiated TPT.

Seven informants responded to the separate survey on potential items for an international TBI registry. Indication of screening (6 out of 7 votes), age category (6/7), TPT initiated (6/7) country of birth, IGRA result positive or negative (5/7), and TPT started (4/7). Data on all candidate variables are presented in the supplementary data (S4). Comments on possibilities and preconditions for a common registry are listed in supplementary data (S1).

## From experience: strengths and barriers in the management of TBI in NDPHS countries and Ukraine

The most commonly reported challenges in care for TBI were the lack of training (four countries) and organisational differences between regions (three countries). Shortage of medicines, shortage of clinical specialists, laboratory testing capacities and stigma were reported by two countries. The informants were also requested to share advice and best practices. Collaboration with other specialists to implement testing for TBI before anti-TNF treatment was mentioned, as well as targeted testing for immigrants and asylum seekers, in order to test only those who would be candidates for TPT, implementation of screening for TBI in contact tracing practices, and online information for the public and for healthcare professionals.

The country representative of Ukraine mentioned the country’s active practice of diagnosing TBI among risk groups, and the utilization of short preventive regimens with rifapentine, including fixed doses. Ukraine stated that despite the military conditions, innovations are actively implemented to find the best practices for Ukrainians to protect them from TB. The self-reported challenges and strengths are presented as a supplementary document (S2).

## Discussion

Here, we report the similarities and differences in the management of TBI in NDPHS countries and Ukraine. Our data indicate that practices of screening, treatment and surveillance of TBI vary considerably throughout the region. In line with previous reports, our data demonstrate that the relatively high incidence of TB in war-affected Ukraine, particularly the high rates of MDR-TB, has a considerable impact on the epidemiology of TB in the region through migration [18, 19]. 

Recently, the PASS to End TB-initiative expressed that in the European region, the prevention of TB can be enhanced through identifying populations for TPT, using algorithms to detect TBI while ruling out disease in cases of infection, and by optimizing TPT regimens [20]. 

In low-incidence countries, the screening of TB among immigrants is mainly voluntary and directed at excluding TB disease through routine chest X-ray among refugees and immigrants from high-incidence countries. IGRA is more commonly applied in young patients, especially in children, as they are considered at risk of developing TB disease. A systematic review found that the diagnostic yield of detecting TB disease among migrants ranged widely by TB incidence in the country of origin, and cost-effectiveness was highest when testing was directed at migrants originating from high TB incidence countries [21]. In Finland, coverage of screening for pulmonary TB was 71.6% when the influx of asylum seekers exceeded expectations and reserved capacity of healthcare services, indicating that TB control management should include preparedness for rapidly changing conditions [22]. Current evidence suggests that the utilization of IGRA in screening for TBI is cost-effective in most high-income countries, especially when directed at populations at highest risk [23]. 

In 2014, the Russian Federation temporarily occupied and annexed Crimea, which marked the start of the current war. In February 2022, Russia launched a full-scale war against Ukraine, while healthcare facilities are often targeted by bombing and rocket attacks, and TB services are disrupted [24]. In this study, the war in Ukraine has notable effects on the epidemiology of TB and especially MDR-TB in other European countries, which is in line with findings by others [14]. A survey among 30 EU/EEA and 13 other European countries identified that TB notification rates among Ukrainians were lower than the expected rate, but higher in host countries that recommend screening, such as Norway, indicating a mismatch between offered health services and TB likelihood [14]. Thus, although beyond the scope of this study, optimizing screening for Ukrainian individuals in Europe could help control the TB epidemic, particularly the spread of MDR-TB. Furthermore, joint efforts and support from abroad to support the healthcare system within the Ukrainian territory are warranted.

Cross-border mobility of Ukrainians and other migrant populations may complicate the follow-up after the diagnosis or during the treatment of TBI or TB disease. However, data on the mobility of migrants and its impact on treatment of TB is scarce. One study highlighted that completion rates of TBI treatment among migrants in Europe is only 54% [25]. Migrants’ access to preventive measures, including diagnosis and treatment of TBI, is essential for cross-border TB control.

In 2024, the WHO published the updated guideline on TPT, including the important recommendation to treat contacts exposed to MDR/RR-TB with levofloxacin as TPT, based on preliminary findings from the TB-CHAMP trial and the VQUIN MDR trial [6, 26]. Our data indicate that in most countries, this new recommendation was not yet implemented at the time of the data collection, in autumn 2024. For other TBI, the regimens commonly applied in the network countries are aligned with the updated WHO guideline, although rifampicin daily for 4 months (utilized in 8/11 countries), and isoniazid combined with rifapentine (utilized in 4/11 countries) are considered alternative options [6]. According to the recommendations of Centers for Diseases Control and Prevention (CDC), short courses of 3–4 months are preferred over 6–9 months of isoniazid monotherapy [17]. Of note, rifapentine was not available in 5/11 countries, as addressed previously by others [27]. When available, rifapentine in combination with isoniazid could provide an alternative option for a 1-month regimen with a non-inferior outcome in comparison to 9 months of isoniazid, and high completion rates in specific populations [28, 29]. 

Performing systematic TBI screening and treatment requires continuous monitoring to evaluate its effectiveness and identify areas for improvement. A national TBI registry was in place in only two countries and covered only TPT. In this study, we also explored attitudes towards a possible international TBI registry. While practical options were proposed, such as a TBI registry as an extension of the TB registry, reservations existed regarding the legislation and the complexity of collecting the data. Cross-border exchange of health data can be challenged by legal restrictions, but this was beyond the scope of our survey. Furthermore, due to the social and political vulnerability of refugees and other migrants, it must be ensured that such a registry cannot be used against the interests of the registered individuals. Our findings indicate the need to define goals, priorities, and the structure of a TBI registry at both national and international levels.

The global response to TB faces enormous acute challenges. The recent withdrawal of US support for international health projects may have disruptive effects on the TB epidemic worldwide [30]. In low TB incidence countries, this may be reflected by rates of imported TB, including MDR-TB, over time.

This study has several limitations. First, despite their geographical closeness, the countries within the NDPHS network display wide variance in characteristics of TB and MDR-TB epidemiology and in the management of TBI. Countries beyond this network may differ in these and other characteristics. This means that the data cannot be extrapolated. Second, the country-specific data is obtained from TB representatives from national TB services, leaving gaps between policies or recommendations and practice possibly undetected. A future study should focus on healthcare workers in TB clinics and those working with high-risk patients to identify additional practical issues possibly overlooked in this study. Third, this study was performed shortly after the publication of the updated WHO guidelines on TPT. Therefore, the implementation of these updated guidelines should be readdressed in future studies. Lastly, data from Ukraine should be interpreted with caution, as they were collected using a modified methodological approach and during an ongoing armed conflict, which may affect completeness and comparability.

In conclusion, this study shows that even within a relatively small geographic region, there are important differences in TB epidemiology, and that the invasion by the Russian Federation in Ukraine has had notable effects on TB incidence, especially of MDR cases, through the mobility of people. Alignment of national strategies with WHO guidelines and adaptation to new challenges within the countries and internationally are warranted for TB control on the national, regional, and global levels. Regional collaboration could focus on improving cross-border TB and TBI registries, increasing access to rifapentine-based regimens, and ensuring adequate screening for high-risk migrant populations.

## Supplementary Information

Below is the link to the electronic supplementary material.

Supplementary Material 1

## Data Availability

Data is available upon reasonable request.
