# Supplementary material for: Screening and treatment practices for tuberculosis infection in Nordic, Baltic and Central European countries and Ukraine in 2023
Source: Eur J Clin Microbiol Infect Dis. 2026 Mar 18;45(6):1827–35. doi: 10.1007/s10096-026-05471-y (PMC13319904; doi:10.1007/s10096-026-05471-y)

Supplementary data

S1. Comments on a potential international TBI registry from the country interviews

	If WHO, ECDC makes a strong recommendation to put up a registry, countries can do it. It is not a problem to add TBI in TB registry, but a legal basis is needed.
	In theory, it would be possible to have a supranational registry for TBI in ECDC countries, due to data security (GDPR) it might be difficult.
	It would be important to assess what additional value a TBI register gives at a country level. In ideal case, the same tool should be used for the whole Europe.
	Is TBI a subject to registration? It is not a contagious condition → assessed as not a serious public health risk → but it may become a public health risk if TBI progresses to TB disease.
	The register should be as simple as possible and have max. 10 common variables to be reported from each country.
	One should assess what are the aims and which part/parts to prioritize. Experience with the TBI treatment registry is that there is a lot of work to get all case-based data reported by the clinicians.
	If there would be better tests, the data for the registry could be collected from the laboratory. Easy general digital system to extract the data form is needed to get TBI data.
	TBI could be included in the general registry on infectious diseases based on laboratory notification of IGRA result. Ordering of IGRA tests should be structured, so that focus groups for testing can be reported and results evaluated.
	The data for some of the variables not chosen could potentially be collected from other sources (medication/prescription registries, TB registries etc.). Challenging to include indicators that requires a certain follow-up time.
	it is difficult to achieve case reporting in my country of something that is not (yet) a disease but aggregated data from the TB clinics could be an option and interesting and motivating for the clinic as a quality check
	Ideally this would be part of our national infectious diseases register so that there is no need to collect data separately e.g. patient date of birth, country of origin, comorbidities etc.
	If to start register, there should be name and ID code. Then patient group -is it indication for screening? (family contact, HIV-pos, before biological treatment etc)<br>Usual test for LTBI in our country is IGRA. There should be option- other test.<br>If register has case based data with ID, later linking to TB registry those cases who fall ill in TB later, can be found there. It can be years later.

Supplementary data

S2: self-reported barriers and strengths (NDPHS and Ukraine). MDR-TB: Multidrug resistant tuberculosis; PLWH: people living with HIV; TBI: tuberculosis infection; TPT: tuberculosis preventive treatment; Answers were slightly redacted for consistent use of language.

**Barriers or challenges**	
Lack of trainings	4 countries
Organizational differences between regions	3 countries
Shortage of clinical specialists	2 countries
Shortage of medicines	2 countries
Laboratory testing capacity is not available in every part of the country	2 countries
Stigma	1 country
Absence of common guidelines	1 country
Other:	
• Rifapentine not available<br>• Knowledge gap in clinicians<br>• Reluctance of parents<br>• Costs and coverage of pediatric care<br>• High MDR-TB rate in the country<br>• It is necessary to improve PLHIV education about LTBI and those who start TPT adherence, dialysis patients (people with end-stage renal disease) and patients preparing for transplantation	each actively mentioned by a country representative
**Self-reported good practices in the management of TBI in the country**	
• TBI testing of immigrants and asylum seekers is targeted so that one should only test those that are candidates for preventive treatment if positive. This was changed in 2016, prior to that everyone in this group were tested for TBI, regardless of individual risk.	Norway
• see information provided through https://explaintb.org/en	Germany
• Good rather systematic testing before immunosuppressive treatment	Denmark
• TB guidance online re screening is not available in English: https://assets.ctfassets.net/8k0h54kbe6bj/7Jj81uoGxSwhTnUPAkPmNN/ce3a2aa24011431be7e8b965d354b986/Lei_beiningar_um_berklaskimun_og_-rakningu_fyrir_heilbrig_isstarfsmenn_2024.pdf<br>• This does not specify details of regimens	Iceland
• TBI-screening is routine and well implemented in contact tracing practices	Finland
• Good collaboration in diagnosis of TBI between pulmonologists and other specialists before anti-TNF treatment.	Latvia

Supplementary data

S3: Topics covered in the country specific interviews (NDPHS countries) 20.11.2024

- Does your country have a National Tuberculosis Program and does it include a chapter or guideline on latent tuberculosis infection (TBI)?
- Is there any TBI information for patients on webpages, in English?
- Are age limits applied in IGRA-screenings, and what are cut-off values for IGRA positive result for TPT?
- Is there consistent use of ICD codes for TBI (e.g. Z22.7)?
- What term for TBI is currently used (TBI or TBI)?
- Are there any experiences of using 1 month daily HP regimen (those countries using it)?
- TPT for MDR-TB contacts, experiences of levofloxacin /plans to start it?
- Ukrainian refugees/asylum seekers, screening practices of TB/TBI, any TPT?
- Can the informant identify good TBI practices? Provide details.
- What are barriers or challenges in carrying out TBI practices? Any details, solutions?
- any regional follow-up of TBI testing and treatment practices?
- Does the informant have any comments or suggestions on LTBI registry (common structure, data to be collected)?
- Are there any plans of building up a national TBI registry? What is the time plan?

Supplementary data

S4.

Question: Which variables or data would be most important to collect in a TBI registry? Please choose 10 options from the list. Answers from 7/10 countries.

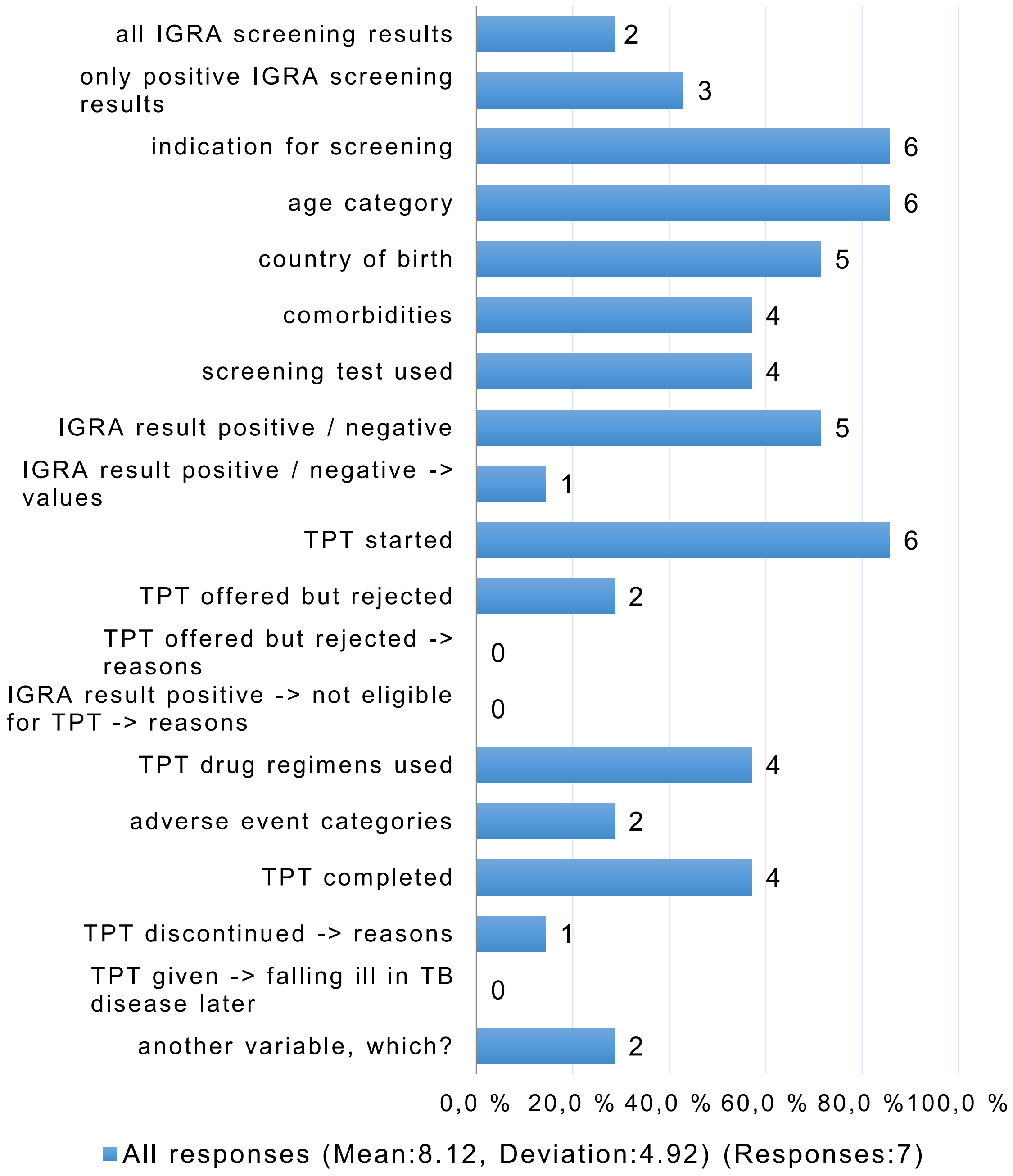

Supplement: Supplementary file 1 — Supplementary Material 1 [file 10096_2026_5471_MOESM1_ESM.pdf]
